# Control of neural crest induction by MarvelD3-mediated attenuation of JNK signalling

**DOI:** 10.1038/s41598-018-19579-5

**Published:** 2018-01-19

**Authors:** Barbara Vacca, Elena Sanchez-Heras, Emily Steed, Sophie L. Busson, Maria S. Balda, Shin-Ichi Ohnuma, Noriaki Sasai, Roberto Mayor, Karl Matter

**Affiliations:** 1 Institute of Ophthalmology, University College London, London, EC1V 9EL UK; 2 Developmental Biomedical Science, Graduate School of Biological Sciences, Nara Institute of Science and Technology (NAIST), 8916-5 Takayama-cho, Ikoma 630-0192, Japan; 3 Department of Cell and Developmental Biology, University College London, Gower Street, London, WC1E 6BT UK; 4 Present Address: Institute of Epigenetics and Stem Cells, Helmholtz Zentrum München, D-81377 Munich, Germany

## Abstract

Tight junctions are required for the formation of tissue barriers and function as suppressors of signalling mechanisms that control gene expression and cell behaviour; however, little is known about the physiological and developmental importance of such signalling functions. Here, we demonstrate that depletion of MarvelD3, a transmembrane protein of tight junctions, disrupts neural crest formation and, consequently, development of neural crest-derived tissues during *Xenopus* embryogenesis. Using embryos and explant cultures combined with a small molecule inhibitor or mutant mRNAs, we show that MarvelD3 is required to attenuate JNK signalling during neural crest induction and that inhibition of JNK pathway activation is sufficient to rescue the phenotype induced by MarvelD3 depletion. Direct JNK stimulation disrupts neural crest development, supporting the importance of negative regulation of JNK. Our data identify the junctional protein MarvelD3 as an essential regulator of early vertebrate development and neural crest induction and, thereby, link tight junctions to the control and timing of JNK signalling during early development.

## Introduction

Tight junctions are multi-protein assemblies essential for epithelia and endothelia to separate compartments of different composition, as they constitute semipermeable paracellular barriers^1^. Tight junctions also serve as bidirectional hubs that receive signals from the cell interior to regulate junctional functions and transmit signals to the cell to guide various cellular processes including gene expression, cell proliferation, migration and survival^1,2^. Junctional signalling mechanisms that regulate cell behaviour are generally inhibitory: They suppress the activity of signalling pathways that promote proliferation and/or migration, such as the JNK (c-Jun N-terminal Kinase) pathway or proliferation-promoting transcriptional pathways involving AP1, ZONAB or YAP/TAZ^3,4^. While loss-of-function *in vivo* studies have led to considerable insights into the roles of junctional barriers in tissue and organ function, the relevance of junctional signalling mechanisms in physiological and developmental processes and the inhibitory role of tight junctions in the regulation of intracellular signalling pathways *in vivo* are poorly understood.

Tight junctions are composed of a large number of transmembrane and cytoplasmic plaque proteins. The cytoplasmic plaque contains a variety of molecules that function as multivalent adaptors and cytoskeletal linkers, and/or as components of signalling mechanisms. Among the transmembrane proteins, occludin, tricellulin and MarvelD3 share a common MARVEL (MAL and related proteins for vesicle traffic and membrane link) domain that differentiates them from the other junctional tetraspan transmembrane proteins of the claudin family^2,5,6^. Claudins are thought to form the junctional barrier and to mediate selective paracellular permeability, which is supported by *in vitro* and *in vivo* studies^7,8^. While the junctional MARVEL domain proteins may modulate junctional permeability properties, they are thought to function mostly as regulators of tight junctions or components of signalling mechanisms by which tight junctions signal to the cell interior^6,9,10^. Nevertheless, the importance of the MARVEL domain proteins as well as other tight junction transmembrane components for the regulation of signalling mechanisms *in vivo* and the physiological relevance of such processes is unclear.

MarvelD3 is the most recently identified junctional tetraspan protein, and loss-of-function experiments demonstrated that it is not required for the formation of functional paracellular barriers^6,11^. Further *in vitro* experiments demonstrated that MarvelD3 is a junctional signalling inhibitor that regulates the epithelial stress response by attenuating JNK activity and, thereby, guides gene expression, cell migration, survival, and proliferation^10^. Similar to other tight junction proteins, MarvelD3 is widely expressed and, typical for MARVEL domain proteins, restricted to vertebrates^6,11^. Despite their wide tissue expression profile, tight junction proteins often serve functions that are important for specific tissues and organs. Tricellulin, for example, is essential for hearing but of limited importance in other tissues^12^. We hence asked whether MarvelD3 is important for specific physiological processes *in vivo*. In a recent study, we found that MarvelD3 regulates eye morphogenesis in *Xenopus laevis*^13^. Intriguingly, while this activity involves JNK, it does not require an inhibitory but an as yet ill-defined stimulatory function of MarvelD3 in JNK signalling.

Here, we asked if MarvelD3 and its suppressive function in JNK signalling are required for dynamic processes during development. Early development relies on interplay between gene expression, cell proliferation and cell migration, processes that can be regulated by JNK signalling and require precisely timed regulation of critical signalling mechanisms. Therefore, we focused on the role of MarvelD3 in early developmental processes. Our results show that MarvelD3 is indeed essential for early development and, specifically, for neural crest induction. We further demonstrate that the underlying mechanism is attenuation of JNK signalling, identifying an unexpected importance of suppression of JNK to enable normal neural crest induction and demonstrating the physiological relevance of tight junction proteins as suppressors of intracellular signalling.

## Results

### Temporal expression of MarvelD3

**Figure 1:**
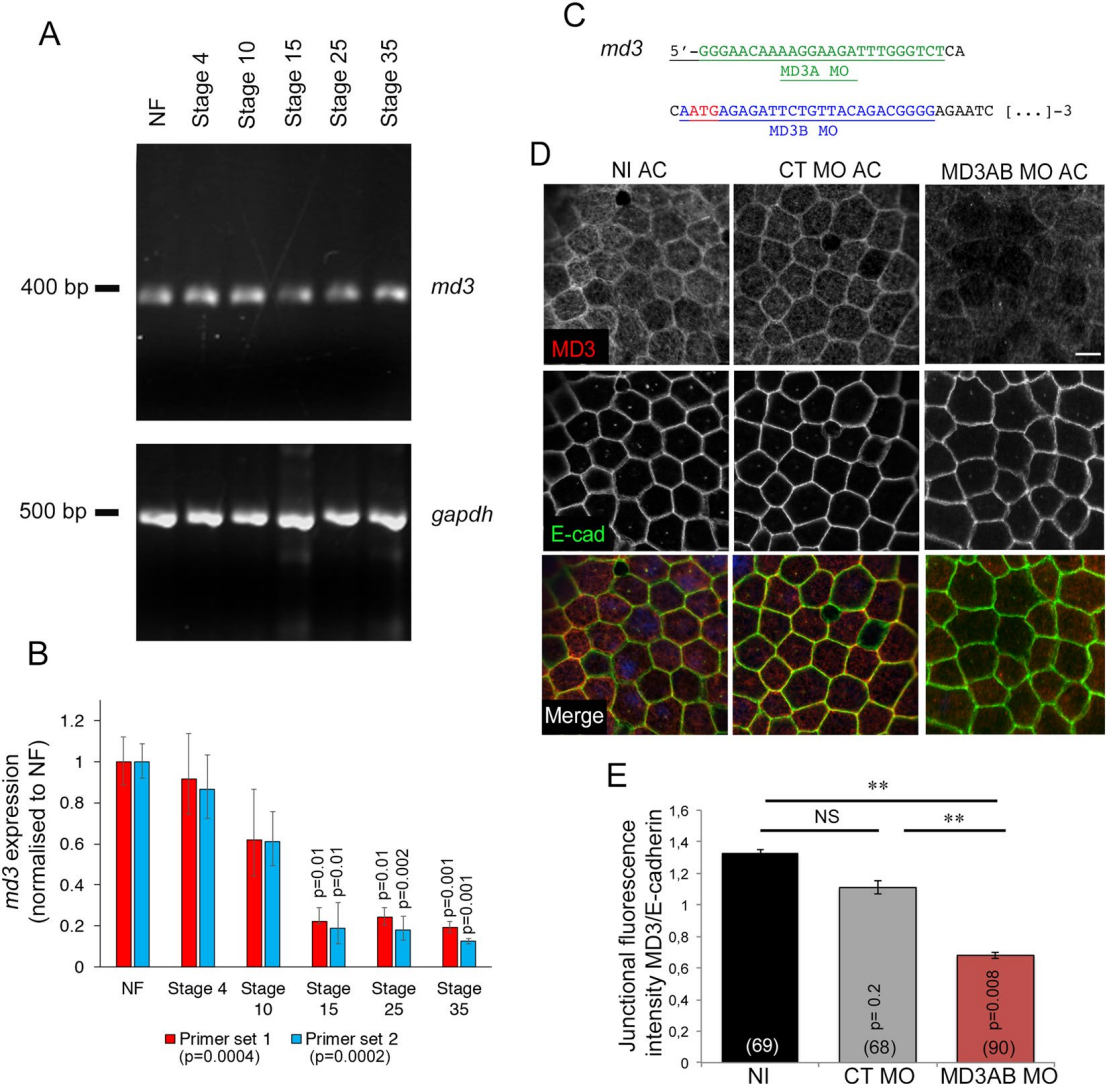
*Marveld3* expression during *Xenopus* embryogenesis and morpholinos efficiency. (**A**) Analysis of *marveld3* transcript expression by semi-quantitative RT-PCR in non-fertilized eggs (NF) and whole embryos from stage (St) 4 to 35; *gapdh* expression was used as a positive control; bp: base pairs. (**B**) Quantification of *marvelD3* expression levels. Quantitative PCR was performed with two different sets of primers for *marvelD3* and a pair of primers amplifying *odc* as a normaliser. Shown are means ± 1 SD of measurements with three independent mRNA isolations per developmental stage. ANOVA values are provided in the graph legend. The indicated p-values in the graph were calculated with t-tests comparing to the corresponding NF values. (**C**) MD3A (green) and MD3B (blue) morpholino sequences are indicated in the 5′-end of the *Xenopus marveld3* sequence; the start codon is indicated in red. (**D**) Analysis of MarvelD3 depletion by immunofluorescence was performed in animal caps derived from stage 8 embryos that had been injected with control or MD3AB morpholinos into both blastomeres at the 2-cell stage. MarvelD3 (red) and E-cadherin (adherens junction marker; green) expression was analyzed by immunofluorescence in animal caps explants. Scale bar, 100 μm; NI, non-injected embryos. (**E**) Ratio of fluorescence intensity at junctions for MarvelD3 and E-cadherin. Mann Whitney test p values are on the graph; the number of cells counted is indicated in brackets on the graph bars; black bar, NI animal caps; grey bar, control morpholino- and red bar, MD3AB morpholino-injected animal caps; red bar, MD3AB morpholino-injected animal caps.

Tight junction components of maternal origin are assembled into adhesive complexes as early as the first embryonic cleavage and are gradually replaced by their zygotic counterpart during mid-blastula transition^14,15^. Thus, we asked whether and when *marveld3* transcript is expressed during early *Xenopus* embryogenesis. The temporal distribution of *marveld3* expression was determined by RT-PCR in non-fertilized eggs (NF) and blastula (stage 3) to identify the maternal RNA and in gastrula (stage 10, gastrulation), neurula (stage 15, neurulation), tailbud (stage 25, convergent extension) and tadpole (stage 35, organogenesis) for expression of zygotic RNA. We found maternal and zygotic *marveld3* expression compatible with an early developmental function (Fig. 1A). Relative abundance was highest in non-fertilized eggs, suggesting a significant maternal contribution to the mRNA pools during early development (Fig. 1B).

To probe for a function of MarvelD3, we designed two distinct translation blocking morpholino antisense oligonucleotides targeting either, the 5′-untranslated region (MD3A morpholino), or, the beginning of the coding region (MD3B morpholino) of the *marveld3* mRNA (Fig. 1C). Additionally, we used a standard negative control morpholino. First, we tested the efficiency of the morpholinos in downregulating MarvelD3 expression by analysing MarvelD3 protein level in animal cap explants by immunofluorescence. Animal cap explants, corresponding to the ectodermal epithelium were generated by dissecting embryos injected into both blastomeres with morpholinos. MarvelD3 was detected at cell-cell contacts in non-injected and control morpholino animal caps, confirming its association with the epithelial junctional complex as in mammalian cells (Fig. 1D)^6,11^. Animal caps presented a reduced junctional staining of MarvelD3 upon injection of MarvelD3 morpholinos compared to animal caps derived from non-injected and control morpholino-injected embryos (Fig. 1D-E). Thus, expression of junctional MarvelD3 can be effectively reduced. The junctional localization of E-cadherin was not affected by MarvelD3 depletion, and no gross morphological defects were observed in depleted ectodermal epithelium (Fig. 1D). These data indicate that morpholinos designed to target MarvelD3 efficiently reduce the expression of MarvelD3 protein.

### MarvelD3 depletion disrupts neural crest derivative differentiation

**Figure 2:**
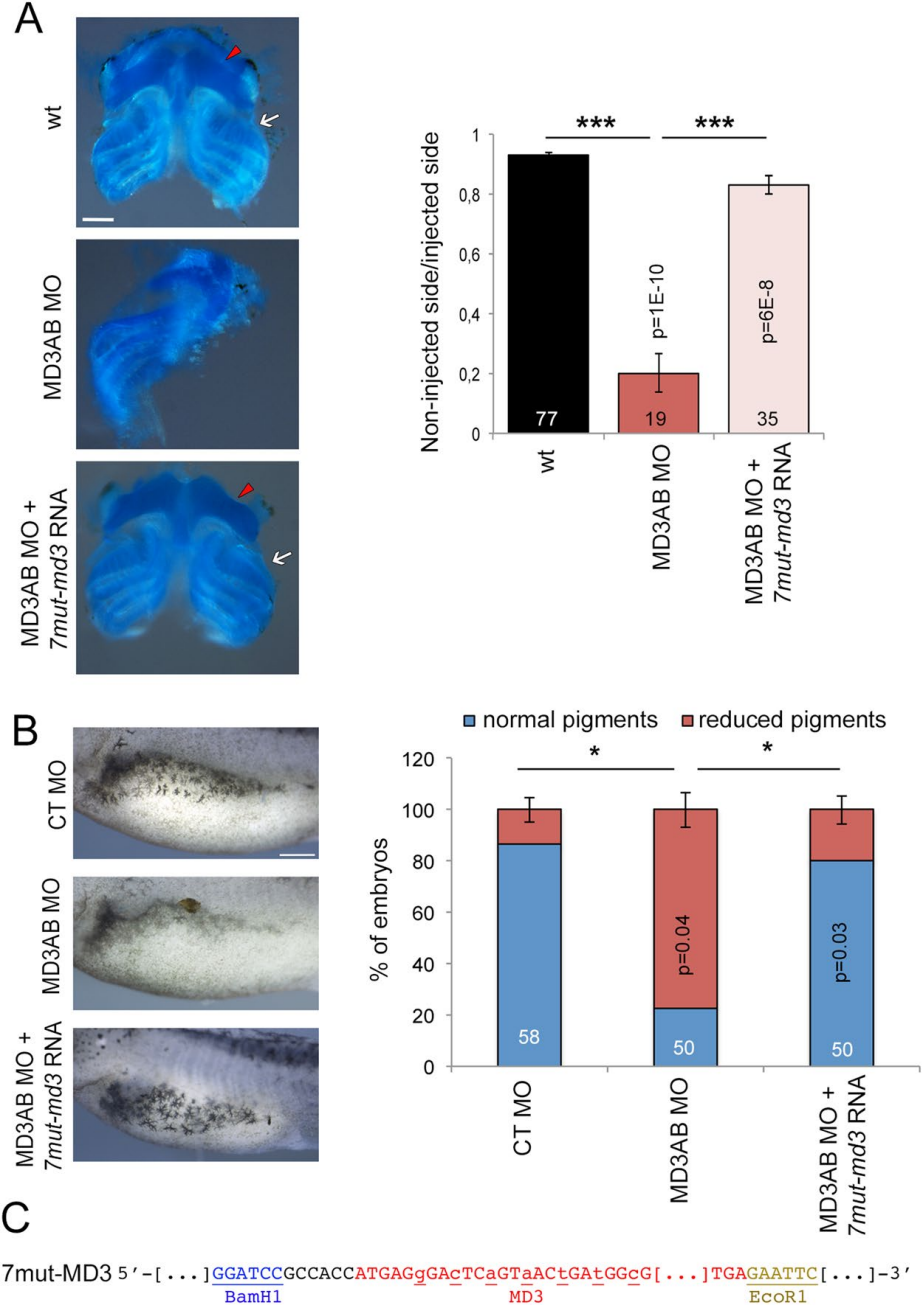
MarvelD3 depletion disrupts neural crest derivatives development. (**A**) Craniofacial cartilage morphology was analysed and its size measured in control, MD3AB morpholino-injected embryos and embryos co-injected with *7mut-marveld3* mRNA. Note, ceratohyal (red arrowhead) and ceratobranchial (white arrow) cartilage was reduced in MarvelD3 morphants. (**B**) The lateral line pigmentation (stage 42) pattern was analysed and quantified in control morpholino, MD3AB morpholino-injected embryos and embryos co-injected with *7mut-marveld3* mRNA. Scale bars, 500 μm; Student t-test p values and the number of embryos analysed are indicated on the graph; blue bar, normal pigments; red bar, reduced pigments; the number craniofacial cartilage samples analysed is indicated on the graph; black bar, wt; red bar, MD3AB morpholinos; light red bar, MD3AB morpholinos + *7mut-marveld3* RNA. (**C**) *7mut-marveld3* was subcloned in pCS2+ vector (BamH1/ EcoR1). Restriction sites: underlined; mutated bases of *marveld3*: underlined lower cases.

In our previous study, MarvelD3 depleted embryos showed no visible morphological alteration until the neurula stage; however, tailbud embryos presented defective head morphology in addition to abnormal eye development^13^. This prompted us to ask whether craniofacial cartilage morphogenesis was affected. Indeed, the structure and size of the craniofacial cartilage were severely disrupted in MarvelD3 morphants (Fig. 2A). The craniofacial cartilage is a neural crest derivative and its abnormal development may reflect a deregulation of processes controlling neural crest formation and/or subsequent neural crest cell migration^16^. Hence, we asked next whether the morphogenesis of a different neural crest derivative was also affected. Figure 2B shows that development of the normal pattern of lateral line pigmentation, formed by neural crest-derived melanocytes, was indeed disturbed upon MarvelD3 morpholino injection, supporting a possible effect of MarvelD3 depletion on neural crest development. We further tested specificity by generating a MarvelD3 encoding mRNA that is not targeted by the two morpholinos. As MD3A morpholino targets the 5′-non-coding region, only the coding region was expressed and seven silent substitutions were introduced into the sequence targeted by MD3B morpholino (*7mut-marveld3* mRNA; Fig. 2C^13^). Craniofacial cartilage morphology and size, as well as lateral line pigmentation were restored by co-injection of *7mut-marveld3* mRNA with MarvelD3 morpholinos, confirming the specificity of the morphant phenotype (Fig. 2B and C). MarvelD3 is thus required for normal development of two distinct neural crest derivatives.

### Ectodermal expression of *marveld3* overlaps with prospective neural crest domain

**Figure 3:**
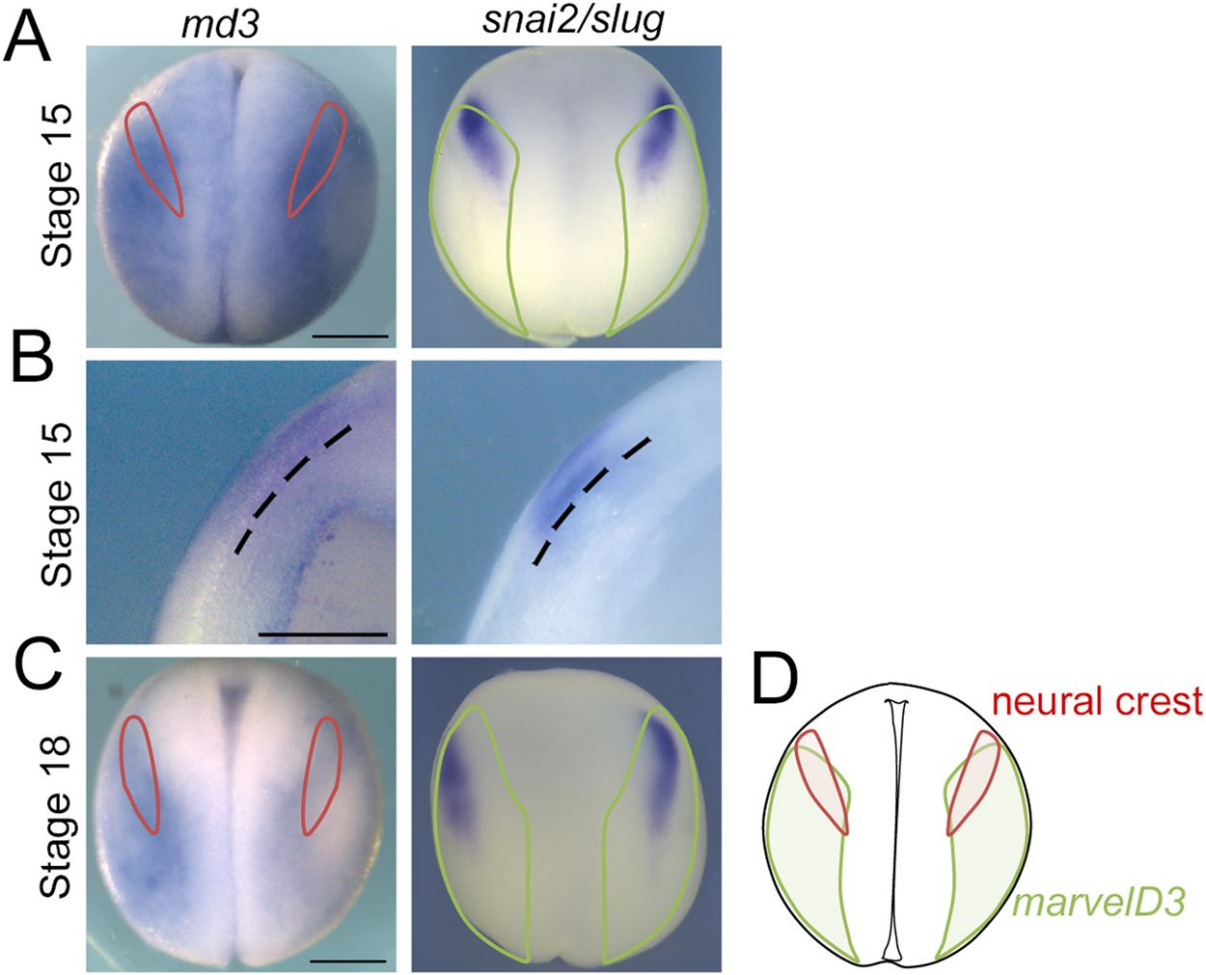
**marveld3** transcript expression domain at neurula stage. Dorsal view of stage 15 (**A**) and 18 (**C**) whole embryos analysed for *marveld3* and *snai2/slug* expression by Whole-mount *in situ* hybridization (WISH). (**B**) WISH analysis of *marveld3* and *snai2/slug* expression on transversal sections of bisected embryos at stage 15. (**D**) Scheme representing the expression domains of *marveld3* (green) and neural crest (red).

As MarvelD3 knockdown disrupts differentiation of two neural crest derivatives, we asked whether *marveld3* is expressed in the ectoderm or mesoderm, both tissues are known to produce signals required for neural crest induction, or whether *marveld3* is expressed in the prospective neural crest domain at the neural plate border, the region between the neural and non-neural ectoderm^16^. To determine the spatial distribution of the *marveld3* transcript, we analysed its expression pattern by whole-mount *in situ* hybridization in whole and bisected embryos. This revealed that *marveld3* is expressed in the dorso-lateral region of the embryo but excluded from the most dorsal area corresponding to the neural plate (Fig. 3A,C). Interestingly, *marveld3* expression extends towards the neural plate border where the neural crest arises, as suggested by the overlap of *marveld3* with the *snai2/slug* territory, an early neural crest marker (Fig. 3A,C,D). Nevertheless, *marveld3* expression is not exclusive to the prospective neural crest domain but has a wider distribution. Cross sections showed that *marveld3* expression is enriched in the superficial ectodermal layer expressing *snai2/slug* (Fig. 3B)^17^. The *marveld3* expression pattern thus supports a potential role in neural crest formation.

### MarvelD3 depletion disrupts the expression pattern of early neural crest markers

**Figure 4:**
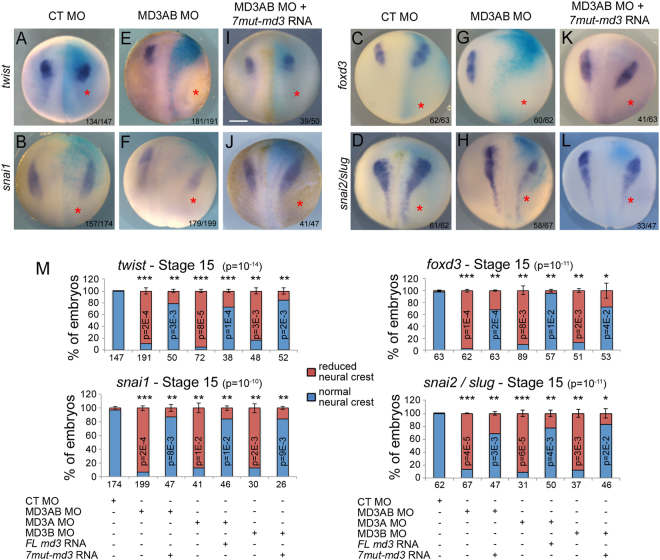
MarvelD3 is required for neural crest formation. Neural crest induction was analysed at stage 15 by WISH using probes against the neural crest markers *twist*, *snai1*, *foxd3* and *snai2/slug*, respectively, in control morpholino (**A**–**D**), MD3AB morpholinos (**E**–**H**) and embryos co-injected with MD3AB morpholino + *7mut-marveld3* RNA (**I**–**L**). The number of embryos analysed is indicated at the bottom right of each panel; red asterisk, injected side of the embryo with the light blue β-galactosidase staining; purple corresponds to the WISH staining; scale bar, 500 µm. (**M**) Quantification of neural crest induction was performed for *twist*, *snai1*, *foxd3* and *snai2/slug*. ANOVA values are provided in parenthesis in the title of each graph. Student t-test p values and the number of embryos analysed are indicated under the graph. See Supplemental Fig. S1 for images of embryos injected with individual MD3A or MD3B morpholinos and corresponding rescue experiments.

Given that *marveld3* expression overlaps with the prospective neural crest domain at the neurula stage, we asked whether MarvelD3 regulates early neural crest formation. We therefore analyzed the impact of MarvelD3 depletion on the expression pattern of the early neural crest markers *twist*, *snai1*, *foxd3* and *snai2/slug*^18^. The expression of these neural crest specifiers was indeed reduced on the MD3AB morpholino-injected side while their expression was unaffected in control morpholino-injected embryos (Fig. 4). A similar reduction of the expression domain of early neural crest markers was obtained by the injection of either MD3A or MD3B morpholino alone (Fig. 4 and Supplemental Fig. S1). As morpholinos targeting different sequences of *marveld3* mRNA reduced expression of neural crest markers, this phenotype is unlikely to have resulted from an off-target effect of the morpholinos. Moreover, co-injection of morpholino-resistant *marveld3* mRNA with both morpholinos together or separately (*7mut-marveld3* mRNA resistant to both morpholinos was used when both or only MD3B morpholino were injected, and *fl marveld3* mRNA corresponding to the coding sequence of marveld3, when MD3A morpholino was injected; Supplemental Fig. S1A) efficiently rescued the disrupted expression of neural crest markers (Fig. 4 and Supplemental Fig. S1). Thus, defective neural crest development observed upon MarvelD3 depletion was induced by morpholinos targeting two different, non-overlapping *marveld3* mRNA sequences and was effectively rescued by non-targeted mRNAs encoding *marveld3*, supporting the specificity of the observed phenotype in neural crest induction.

### MarvelD3 depletion causes displacement of the neural plate border

**Figure 5:**
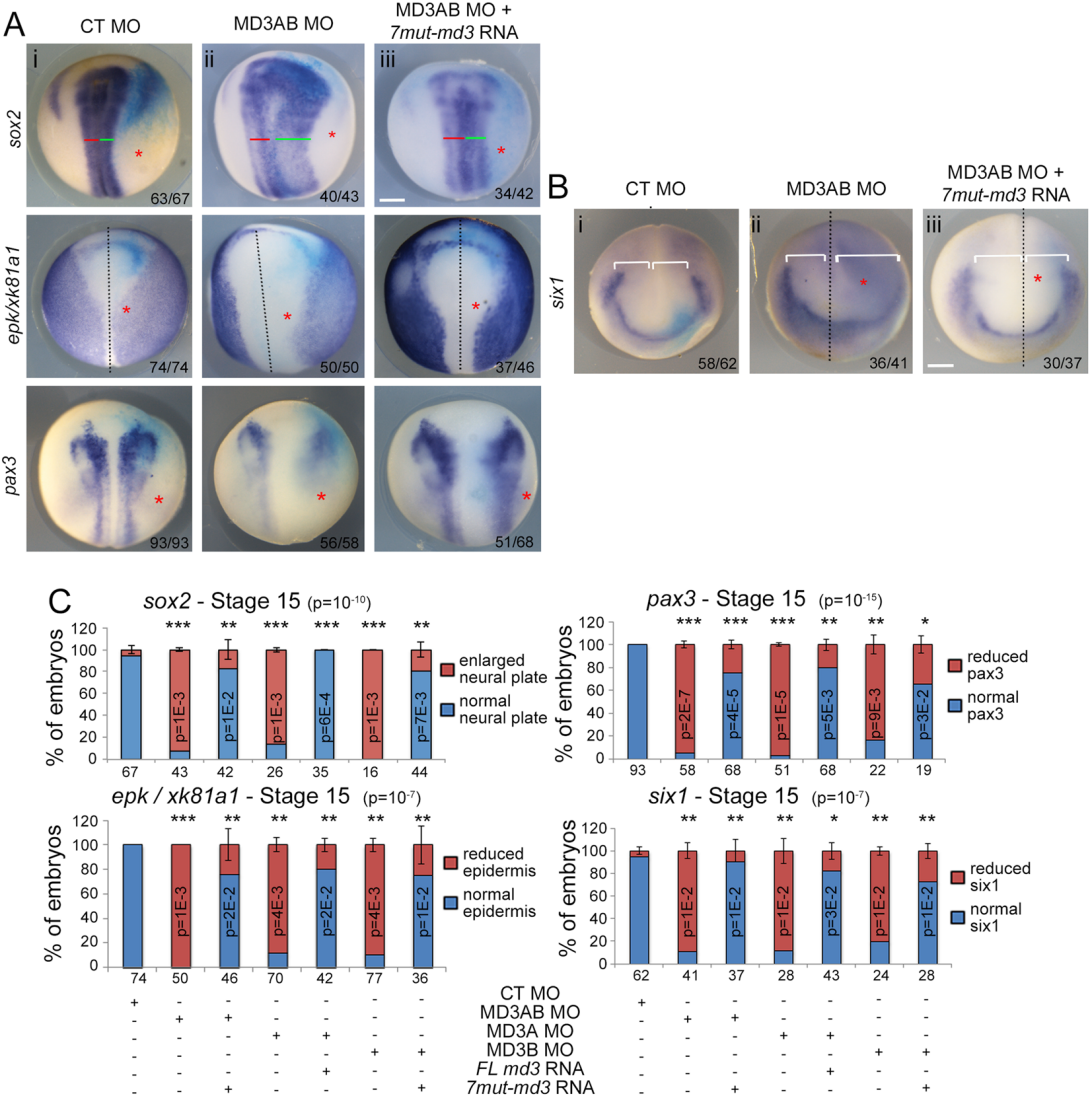
MarvelD3 is required for neural plate border positioning. (**A**) Neural plate border positioning was analysed at stage 15 by WISH with probes against the neural/non-neural ectoderm markers (*sox2*; *epk/xk81a1*) and neural plate border marker *pax3*, while placode formation was analysed with (**B**) a pan-placodal marker, *six1*, in control morpholino- (**A**, i; **B**, i), MD3AB morpholino-injected embryos (**A**, ii; **B**, ii) and embryos co-injected with MD3AB morpholino + *7mut-MarvelD3* mRNA (**A**, iii; **B**, iii). (**A**) Red and green lines demarcate medio-lateral expansion of *sox2*; (**A**,**B**) black doted lines identify the midline; (**B**) white brackets indicate the distance from the midline; the number of embryos analysed is indicated at the bottom right of each panel; red asterisk, injected side of the embryo with the light blue β-galactosidase staining; scale bars, 500 µm. (**C**) Quantification of neural crest induction was performed for *twist*, *snai1*, *foxd3* and *snai2/slug*. ANOVA values are provided in parenthesis in the title of each graph. Student t-test p values and the number of embryos analysed are indicated; blue bar, normal neural plate (NP; *sox2*) or epidermis (*epk/xk81a1*) or neural plate border (*pax3*, *six1*); red bar, enlarged neural plate (*sox2*) or reduced epidermis (*epk/xk81a1*) or reduced neural plate border (*pax3*, *six1*). See Fig. S2 for images of embryos injected with individual MD3A or MD3B morpholinos and corresponding rescue experiments.

The neural crest is formed at the neural plate border; hence, MarvelD3-depleted embryos may not only have defects in early neural crest formation but also in positioning of the neural plate border. This hypothesis is supported by the observation that residual expression of neural crest markers tended to be laterally displaced in MarvelD3 morpholino-injected embryos (Fig. 4). We hence asked whether MarvelD3 knockdown affects expression of *sox2*, a marker for the neural plate; *epidermal keratin* (*epk/xk81a1*), a gene expressed in non-neural ectoderm; and *pax3*, a specifier of the neural plate border. The *sox2* domain was mildly enlarged in the anterior neural plate and thicker posteriorly, while *epk/xk81a1* was reduced in the anterior and dorso-lateral region of MarvelD3 morpholino-injected embryos (Fig. 5A,C and Supplemental Fig. S2A). These results suggest that the neural plate domain is increased at the expense of the non-neural and the neural crest domain. Consistently, *pax3* expression was reduced and posteriorised in MarvelD3 knockdown embryos (Fig. 5A,C and Supplemental Fig. S2). Placodes, which also form at the neural plate border, were mildly enlarged laterally as indicated by the expression of the pan-placodal marker *six1* (Fig. 5B,C and Supplemental Fig. S2). Co-injection of morpholino-resistant *marveld3* mRNA rescued the border positioning phenotype induced by both MarvelD3 morpholinos (Fig. 5 and Supplemental Fig. S2). Depletion of MarvelD3 thus led to a specific deregulation of neural plate border positioning.

### MarvelD3 knockdown disrupts neural crest precursor formation

**Figure 6:**
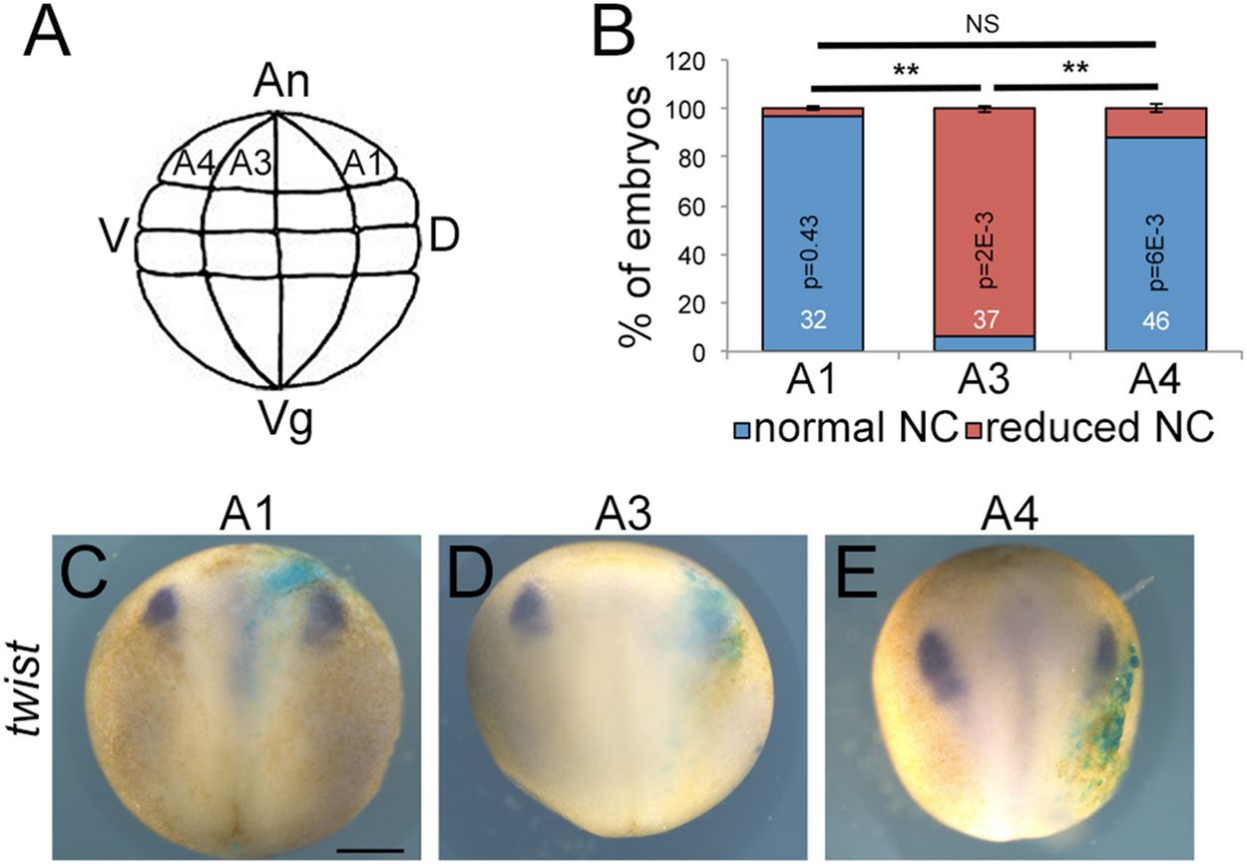
MarvelD3 depletion targeting a neural crest progenitor inhibits *twist* expression. (**A**) Scheme of the lateral view of a 32-cell stage embryo (adapted from Xenbase). The blastomeres are identified with from dorsal (**D**) to ventral (V) as A1 (mainly neural plate), A3 (mainly neural crest and epidermis) and A4 (mainly epidermis); An, animal; Vg, vegetal. (**B**) Neural crest induction (stage 15) was quantified by analysing *twist* expression by WISH in embryos injected at 32-cell stage either in the A1, A3 or A4 blastomere; Student t-test p values and the number of embryos analysed is indicated on the graph; blue bar, normal neural crest; red bar, reduced neural crest. Expression of the neural crest marker, *twist* by WISH in embryos injected in A1 (**C**), A3 (**D**) or A4 (**E**) blastomere, respectively at 32-cell stage. β-galactosidase (light blue staining) is expressed in the cells derived from the targeted blastomere. Note the reduction of *twist* expression only in A3 injected embryos. Scale bar, 500 μm.

As the expression of *marveld3* is not exclusive to the neural crest territory, we asked whether MarvelD3 is directly or indirectly affecting development of neural crest precursors formation by injecting individual blastomeres in 32-cells stage embryos to target neural plate, neural crest, or epidermis^17,19^. The A3 blastomere develops into neural crest and contributes to the epidermis, whereas the A1 and A4 blastomeres are mainly fated to become neural plate and epidermis, respectively^19^. Our result revealed that A1- and A4-injected embryos did not reveal a clear phenotype, indicating that targeting precursors of the neural plate and epidermis is not sufficient to induce the neural crest phenotype. In contrast, a strong reduction of the expression of the neural crest marker in embryos derived from the A3-injected embryos was observed, indicating that the induction of the phenotype required targeting of neural crest precursors (Fig. 6). These data thus suggest that the disruption of neural crest is a direct consequence of MarvelD3 depletion in cells that give raise to the neural crest.

### MarvelD3 is required for neural crest induction

**Figure 7:**
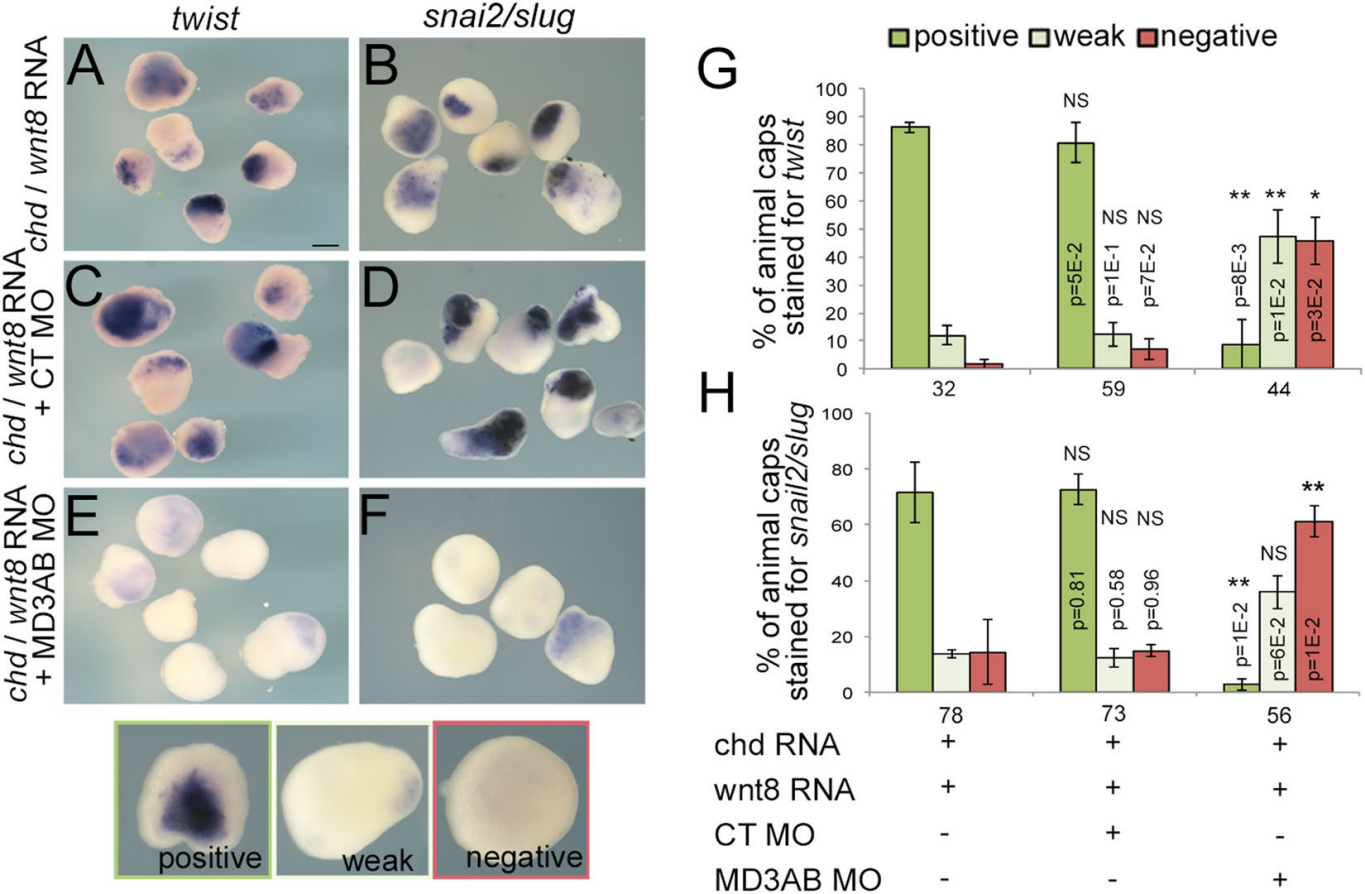
MarvelD3 is required for neural crest induction. Expression of the neural crest markers *twist* and *snai2/slug* during neural crest induction (stage 15) was detected by WISH in animal caps explants injected with *chd* mRNA + *wnt8* mRNA alone (**A**,**B**) and co-injected with control (**C**,**D**) or MD3AB morpholinos (**E**,**F**); scale bar, 500 μm. Neural crest induction phenotype was quantified in animal caps and classified as positive (dark green), weak (light green) and negative (red) for *twist* (**G**) and *snai2/slug* (**H**) expression. Student t-test p values and the number of animal caps analysed are indicated.

MarvelD3 depletion in neural crest precursor cells reduces the expression of neural crest marker as early as stage 15, suggesting MarvelD3 might be required during neural crest induction. The neural crest is induced by a combination of signals secreted by the ectoderm as well as the mesoderm, such as wnt8 and chordin, an antagonist of bone morphogenetic protein (BMP)^16^. To test whether MarvelD3 might modulate such signals, we stimulated neural crest induction in animal cap explants by co-injecting *wnt8* and *chd* mRNA; these mRNAs induce neural crest fate as monitored by *twist* and *snai2/slug* expression (Fig. 7A,B,G,H)^20^. To determine the role of MarvelD3, neural crest inducers were co-injected with control or MarvelD3 morpholinos (Fig. 7C–H). Figure 7 shows that injection with MarvelD3 morpholinos, but not control morpholino, led to a strong attenuation of neural crest induction in animal caps as only weak signals for *twist* and for *snai2/slug* expression were observed. MarvelD3 thus plays an essential role in enabling signals inducing neural crest.

Mesodermal defects can affect neural crest induction^21^. Therefore, we asked whether MarvelD3 also regulates mesoderm formation. We analysed the expression of two mesodermal markers, *xbrachyury* (*xbra*) and *goosecoid* (*gsc*) at mid-gastrula stage. Expression of neither of the two mesodermal markers was altered in MarvelD3 morphants (Supplemental Fig. S3), excluding the possibility of a mesodermal contribution to the neural crest phenotype.

### MarvelD3 depletion enhances JNK signalling during neural crest induction

**Figure 8:**
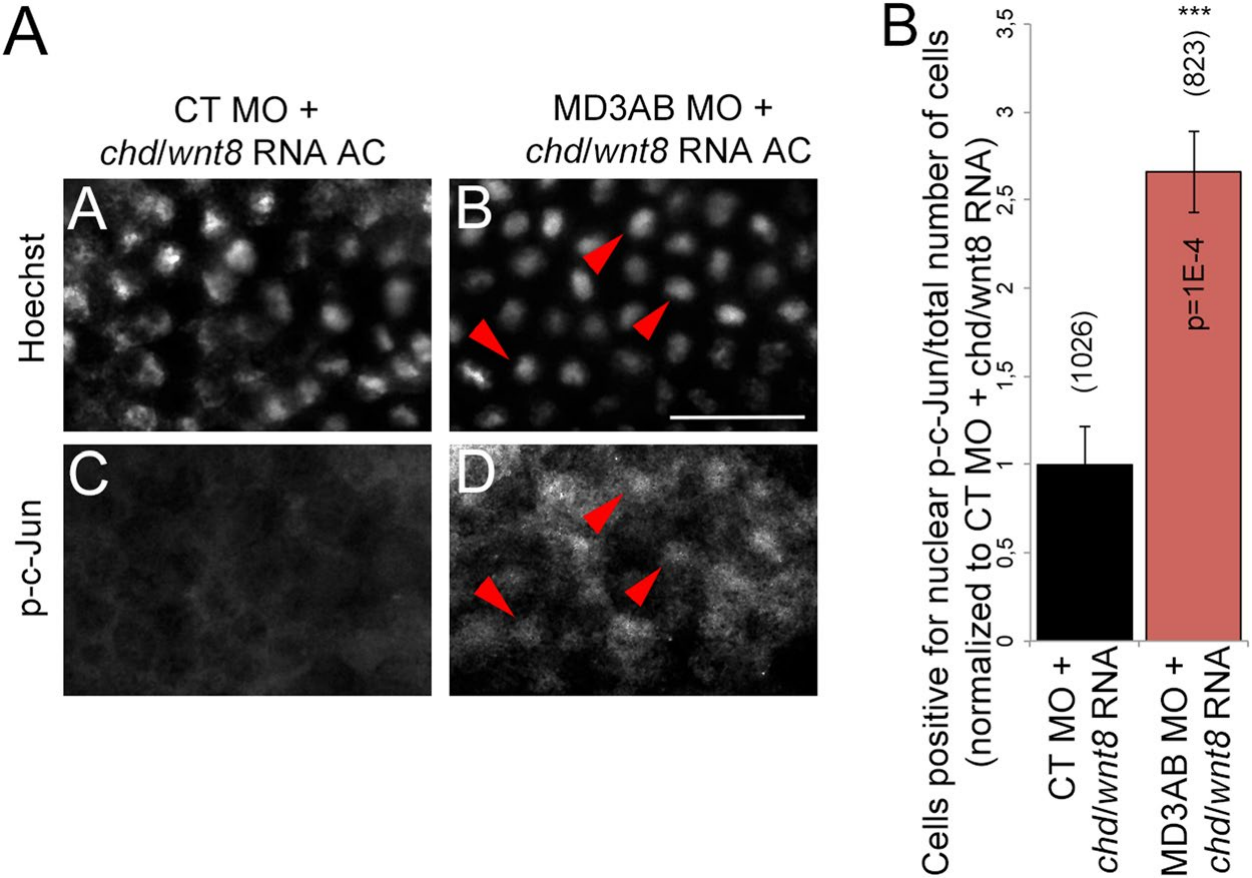
MarvelD3 depletion activates JNK signalling. Analysis of JNK signalling by immunofluorescence using an antibody against phosphorylated form of c-Jun (p-c-Jun) in animal caps injected with *chd* mRNA + *wnt8* mRNA + control morpholino (**A**,**C**) or *chd* mRNA + *wnt8* mRNA + MD3AB morpholinos (**B**,**D**). Nuclei were stained with Hoechst; red arrowheads: examples of nuclear co-localization between Hoechst and p-c-Jun staining; scale bar, 50 μm. (**E**) Quantification of the number of cells with a positive nuclear p-c-Jun staining / total number of cells. Mann-Whitney test p values and the total of cells counted (number in brackets) are indicated on the graph; the values are normalized to control morpholino + *chd* mRNA + *wnt8* mRNA; black bar, *chd* mRNA + *wnt8* mRNA + control morpholino; red bar, *chd* mRNA + *wnt8* mRNA + MD3AB morpholinos.

Neural crest induction requires the coordination of distinct signalling mechanisms that need to be activated in a controlled manner as they can negatively regulate each other. JNK is a crucial component of such signalling pathways and its activity is known to be required after initial induction of neural crest cell differentiation for neural crest cells to migrate to specific sites in the developing embryo. As we previously found that reduced expression of MarvelD3 *in vitro* stimulates JNK signalling and cell migration^10^, we asked whether MarvelD3 depletion leads to precocious activation of JNK during neural crest induction. Thus, we tested whether MarvelD3 knockdown stimulates JNK signalling in the animal cap model by assessing the induction and translocation of phosphorylated c-Jun (p-c-Jun) to the nucleus as a measure for activation of JNK. Figure 8 shows that animal caps derived from MarvelD3 morpholinos-injected embryos exhibited an increased number of cells with a positive nuclear phosphorylated c-Jun staining compared to control morpholino-animal caps. Thus, reduced MarvelD3 expression indeed leads to increased JNK signalling, suggesting that attenuation of JNK activation may be important during neural crest induction.

### Chemical inhibition of JNK rescues neural crest induction in MarvelD3 knockdown

If increased JNK activity is indeed the underlying reason for the observed neural crest induction defect, inhibiting JNK should rescue the phenotype. To test this, we first assessed the effectiveness of the JNK inhibitor SP600125 (0.5 μg.ml^−1^) in animal caps using a constitutively active form of JNK to stimulate the nuclear translocation of the phosphorylated form of c-Jun. *Constitutively active-jnk*-injected animal caps showed an increase in the number of cells positive for nuclear phosphorylated c-Jun, which was attenuated by incubation with SP600125 (Supplemental Fig. S4). SP600125 thus inhibits JNK signalling in *Xenopus*.

**Figure 9:**
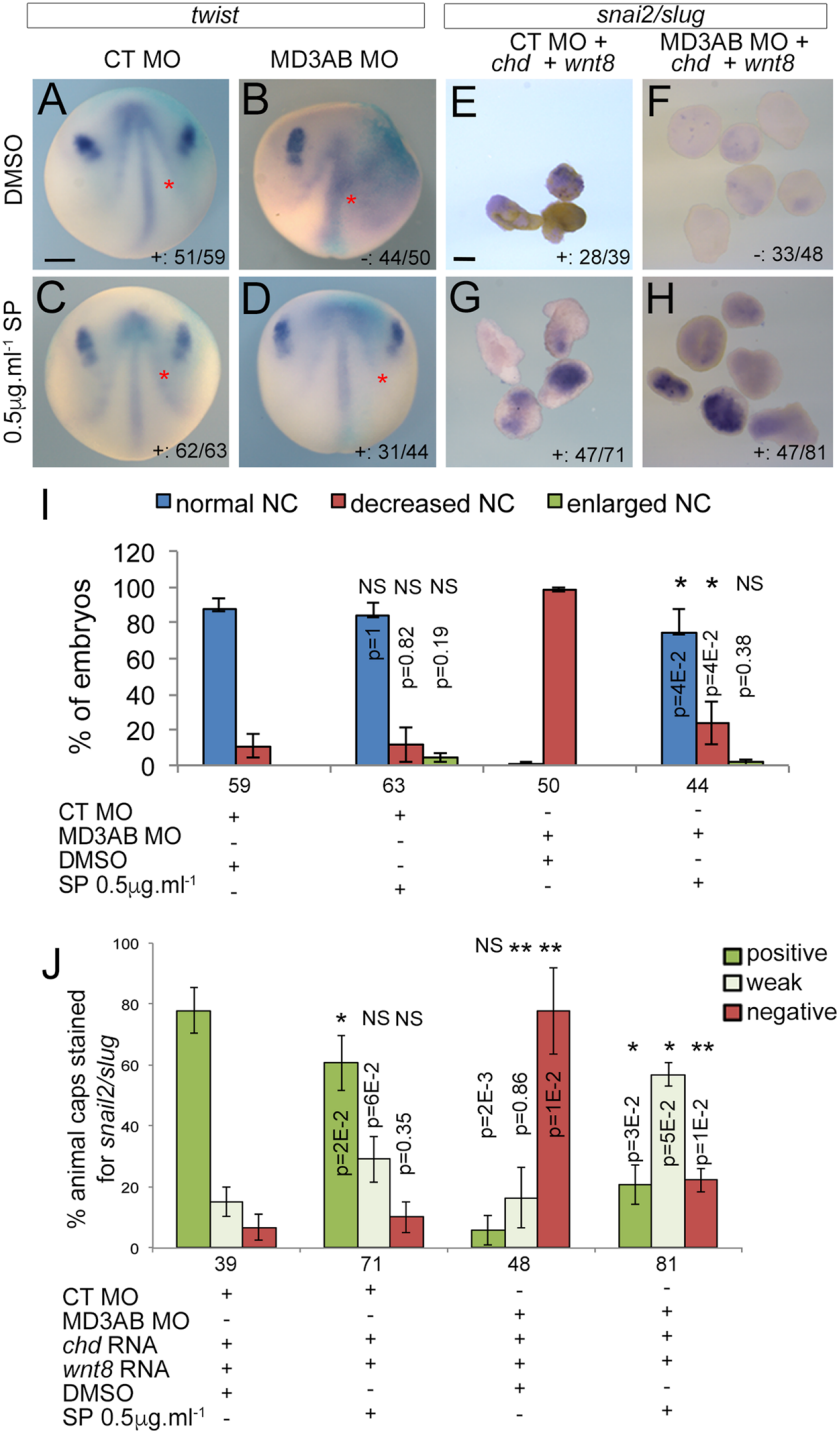
Rescue of neural crest formation by small molecule inhibitor for JNK in MarvelD3 depleted embryos. Neural crest induction (stage 15) was analysed by WISH against *twist* in control and MD3AB morpholino-injected embryos **(A**–**D**; red asterisk, injected side of the embryo with β-galactosidase staining) and against *snai2/slug* in animal caps explants co-injected with *chd* mRNA + *wnt8* mRNA + control morpholino or *chd* mRNA + *wnt8* mRNA + MD3AB morpholinos. Both embryos and animal caps were treated from stage 11 to 15 with DMSO or 0,5 μg.ml^−1^ SP600125, a JNK inhibitor (**E**–**H**). Neural crest induction was quantified according to *twist* or *snai2/slug* expression in embryos (**I**) and animal caps (**J**), respectively. Mann-Whitney test p values and the number of embryos or animal caps analysed are indicated; red asterisk, injected side of the embryo with light blue β-galactosidase staining. (**I**) blue bar, normal neural crest; red bar, reduced neural crest; (**J**) dark green bar, positive; light green bar, weak; red bar, negative *snai2/slug* staining. + , embryos or animal caps positive or weak for *twist* or *snai2/slug* expression; -, embryos or animal caps negative for *twist* or *snai2/slug* expression. Scale bars, 500 μm.

As MarvelD3 knockdown induces increased nuclear phosphorylated c-Jun (Fig. 8), we next asked whether inhibition of JNK by SP600125 is sufficient to rescue neural crest induction by incubating control and MarvelD3 morpholino-injected embryos with 0.5 μg.ml^−1^ of JNK inhibitor from early gastrula onwards. *twist* expression was unaffected in control morpholino embryos (Fig. 9A,C,I) at stage 15, suggesting the inhibition of JNK itself did not alter neural crest induction. Strikingly, SP600125 was sufficient to rescue neural crest induction in MarvelD3 morphants as monitored by *twist* expression (Fig. 9B,D,I). To corroborate these results, we performed the same experiment with the ectopic neural crest induction assay in animal caps. Similarly, to whole embryos, SP600125 rescued neural crest induction, as monitored by *snai2/slug* expression to a significant extent in MarvelD3 morpholino-injected animal caps (Fig. 9E–H,J). Hence, attenuation of the JNK pathway by a small molecule inhibitor is sufficient to rescue neural crest induction in MarvelD3 morphants.

### Attenuation of JNK signalling is required for neural crest induction

**Figure 10:**
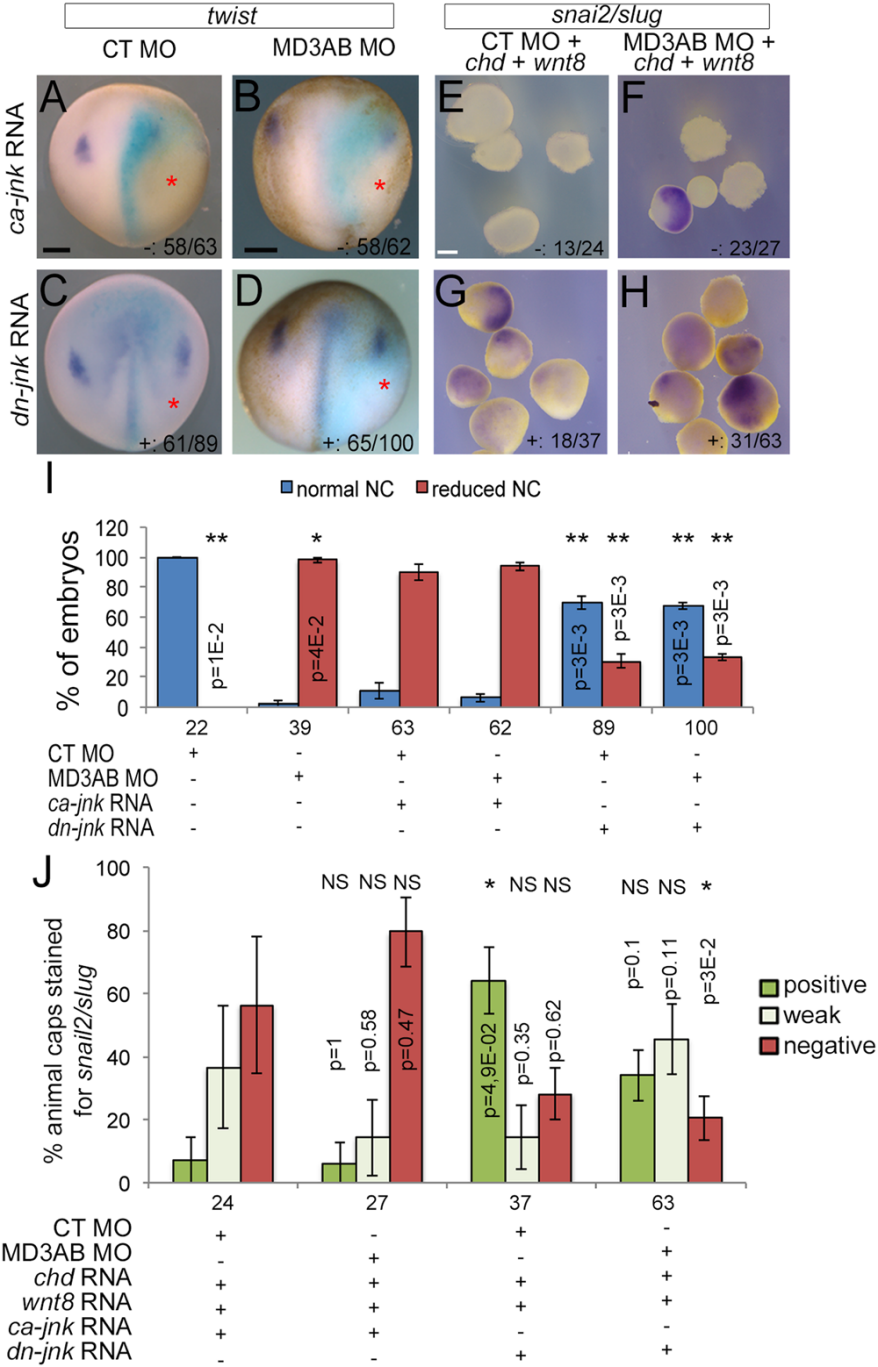
JNK pathway inhibition is required for neural crest formation in MarvelD3 knockdown embryos. Neural crest induction (stage 15) was analysed and quantified by WISH against *twist* in control and MD3AB morpholino embryos co-injected with *constitutively active-jnk* (*ca-jnk*) or *dominant negative-jnk* mRNA (*dn-jnk*) (**A**–**D** and **I**) and against *snai2/slug* in animal caps co-injected with *chd* mRNA + *wnt8* mRNA + control morpholino or *chd* mRNA + *wnt8* mRNA + MD3AB morpholinos and with *constitutively active-jnk* or *dominant negative-jnk* mRNA (**E**–**H** and **J**). Mann-Whitney test p values and the number of embryos or animal caps analysed are indicated; red asterisk, injected side of the embryo with light blue β-galactosidase staining. (**I**) blue bar, normal neural crest; red bar, reduced neural crest; (**J**) dark green bar, positive; light green bar, weak; red bar, negative *snai2/slug* staining. + , embryos or animal caps positive or weak for *twist* or *snai2/slug* expression; -, embryos or animal caps negative for *twist* or *snai2/slug* expression. Scale bars, 500 μm.

To confirm that increased JNK activity suppresses neural crest induction, we co-injected mRNAs encoding either a constitutively active form of JNK or a dominant negative JNK mutant with control or MarvelD3 morpholinos into a dorsal blastomere of 8-cell stage embryos and subsequently analysed neural crest induction by whole-mount *in situ* hybridization. *twist* expression was indeed reduced in control and MarvelD3 morpholinos embryos co-injected with *constitutively active-jnk* mRNA, reflecting an inhibition of neural crest induction; a partial rescue of neural crest induction was observed in MarvelD3 morphants co-injected with *dominant negative-jnk* mRNA similarly to the small molecule inhibitor (Fig. 10A–D and I, and Supplemental Fig. S5). Regulation of JNK activity with *constitutively active-jnk* and *dominant negative-jnk* mRNAs also modulated neural crest induction (*snail2/slug* expression) in the animal cap assay (Fig. 10E–H, J and Supplemental Fig. S5). Thus, increased JNK signalling suppresses neural crest induction, and inhibition of JNK signalling using two different approaches, *dominant negative-jnk* mRNA and the small molecule inhibitor SP600125, was sufficient to rescue neural crest induction in MarvelD3 morphants, indicating that attenuation of JNK activity by MarvelD3 is important during neural crest induction.

## Discussion

Here, we identify the tight junction transmembrane protein MarvelD3 as a regulator of early embryogenesis required for neural crest induction. MarvelD3 guides neural crest formation by attenuating JNK pathway activation, a signalling mechanism important for steps following neural crest cell induction such as migration and development of neural crest-derived tissues^16^. MarvelD3 is downregulated during snail1-induced epithelial-mesenchymal transition, a process that also occurs at the onset of neural crest cell migration^22^. Our data hence support a model in which MarvelD3 is part of a mechanism that prevents precocious JNK signalling before dissolution of tight junctions, a pathway that disrupts neural crest induction if activated prematurely by expression of *constitutively active-jnk* mRNA or MarvelD3 depletion. The physiological importance of tight junctions during development is thus not restricted to the formation of tissue barriers and control of paracellular permeability but extends to guiding the activity of at least one central signalling process, the JNK pathway that drives cell migration and dynamics.

The temporal and spatial expression pattern of *marveld3* is compatible with a functional role during early embryogenesis. *marveld3* expression was detected from the non-fertilized egg to the tadpole stage. The junctional complex assembles during the first embryonic cleavage and MarvelD3 is associated with the junctional complex in the *Xenopus* ectoderm as previously shown for mammalian epithelia^11,15,23–25^. Interestingly, *marveld3* distribution also presents similarities with the *c-jun* expression domain at the tadpole stage^26,27^. These data are in agreement with the involvement of MarvelD3 in neural crest development as a regulator of JNK signalling. Although *marveld3* is more widely expressed and not restricted to the neural crest territory, the spatial distribution of the *marveld3* transcript during embryogenesis substantiates its function in neural crest formation. The *marveld3* transcript is strongly expressed in the dorso-lateral ectoderm of early neurulas; hence, it overlaps with the prospective neural crest territory, consistent with a role of MarvelD3 in the early steps of neural crest morphogenesis. A direct role of MarvelD3 in neural crest induction is indeed supported by targeted injections of blastomeres that recapitulated the neural crest induction phenotype only if the targeted blastomere gives rise to neural crest, and also through the requirement of MarvelD3 for neural crest induction in animal caps. Similarly to tadpoles, *marveld3* expression also partially overlaps with *c-jun* expression at the lateral and posterior neural plate border regions^27^, supporting a model in which MarvelD3 participates in the coordination of c-Jun activity at the neural plate border during neural crest formation. We have previously reported that MarvelD3 can inhibit c-Jun activity *in vitro*^10^. This is compatible with our finding in animal cap explants-induced for neural crest formation demonstrating that depletion of MarvelD3 promotes nuclear translocation of phosphorylated c-Jun, reflecting JNK pathway activation. Although JNK signalling can regulate different transcription factors, suppression of c-Jun activation may thus be an important component of MarvelD3 signalling during neural crest induction.

Since *marveld3* expression overlaps with the neural plate border territory where the neural crest arises, we analysed neural plate border positioning in MarvelD3 knockdown embryos and observed a lateral expansion of the neural plate and a complementarily reduction of the non-neural ectoderm. The defective neural plate border positioning observed in MarvelD3 morphants was paralleled by a lateral displacement of the remaining neural crest markers and a corresponding diffuse appearance of the neural plate border specifier *pax3*. Disruption of *pax3* expression is known to affect both neural plate border formation and subsequently neural crest induction in *Xenopus* and mouse^21^,^28–30^. Although the reduced *pax3* domain in MarvelD3 morphants might contribute to the neural plate border positioning defect, the targeted injections in blastomeres at the 32-cells stage revealed that defective neural crest induction in MarvelD3-depleted embryos was due to a direct effect on neural crest precursors. Indeed, reduced expression of genes, such as, the neural crest-inducing genes *foxd3* and *snai2/slug* not only reduces the neural crest but, similar to what we observed in MarvelD3-depleted embryos, also leads to an expansion of the neural plate^31^. Hence, the displacement of the neural plate border is likely to be a consequence of early defects in neural crest induction.

Neural crest induction involves the tight regulation of synergizing signals^21,32^. Steventon *et al*. described neural crest induction as the result of variations of Wnt and BMP activity levels between early gastrula and early neurula stages^33^. The MarvelD3 requirement for neural crest induction in animal caps-induced for ectopic neural crest formation with *wnt8* and *chd* mRNAs suggests that MarvelD3 impacts on this signalling network that centres on canonical Wnt and BMP signalling. For instance, in zebrafish, the inhibition of canonical Wnt signalling promotes the differentiation of neurons instead of pigments^34^, consistent with the observed expansion of the neural plate, reduced neural crest, and, consequently, reduced pigments in MarvelD3 knockdown embryos.

We previously reported that MarvelD3 suppresses JNK signalling *in vitro*^10,13^. Attenuation of JNK signalling by MarvelD3 is essential during neural crest induction: inhibition of JNK activity rescued neural crest development in MarvelD3 morphants while an increase in JNK activity was detrimental for neural crest development. However, JNK may also contribute to neural crest induction as recent studies suggest an involvement of the non-canonical Wnt/ROR2 pathway in neural crest specification in *Xenopus*^35,36^. It is thus conceivable that MarvelD3 might be involved in the regulation JNK to balance non-canonical Wnt and canonical Wnt signaling; hence, neural crest induction is disrupted due to overstimulation of JNK in MarvelD3 morphants or when JNK is hyperactivated by expression of constitutively active*-jnk* mRNA. Deregulation of the non-canonical Wnt/ROR2 pathway leads to a phenotype with a number of similarities with the one of MarvelD3 knockdown but there are clear differences in expression of neural plate border specifiers; thus, it seems unlikely that MarvelD3 is an integral part of ROR2 signalling. The non-canonical Wnt/planar cell polarity pathway, which also involves JNK signalling, is known to control the convergent extension process^37^ and neural crest migration; however, we did not observe defects on the anterior-posterior axis length as would be expected if that pathway was affected^13^. Moreover, we did also not observe an involvement of the mesoderm, further arguing against an involvement of the non-canonical Wnt/planar cell polarity pathway downstream of MarvelD3.

Our data suggest that modulation of JNK activity during the early phase of neural crest induction is required and mediated by a mechanism linked to epithelial integrity via tight junctions. In cancer cells, *snai1* initiates epithelial-mesenchymal transition and downregulates MarvelD3 expression^22^. Hence, induction of neural crest specifiers such as *snai1* leads to downregulation of MarvelD3 and release of the break on JNK signalling. We thus propose that MarvelD3 is part of a regulatory feedback loop that coordinates JNK activity with neural crest formation during early embryogenesis. Future studies will require tissue-specific knockout approaches to determine which cell type(s) are at the origin of the developmental defects and to analyse crosstalk with other signalling pathways to identify why some cell types are affected by downregulation of MarvelD3 and others are not. Experiments will also need to focus on the regulation of MarvelD3 itself as subcellular trafficking of the protein appears an important part of the associated regulatory mechanism *in vitro*^10^. During eye morphogenesis, MarvelD3 does not function as a suppressor of the JNK pathway as during neural crest development but is required for JNK activation^13^; hence, the molecular, cellular and developmental mechanisms will have to be determined that underlie the regulatory switch from JNK repressor to activator.

## Methods

### Xenopus laevis manipulation

Animals were used according to the UK Home Office regulations and the experimental protocols had been approved by the UCL Institute of Ophthalmology Animal Welfare and Ethical Review Body (PPL 70/6750). The embryos were staged according to^38^. Animal caps, dissected at stage 9, were cultured in Steinberg solution (4.6 mM Tris-HCl, pH7.4; 58 mM NaCl; 0.67 mM KCl; 0.34 mM Ca (NO_3_)_2_; 0.83 mM MgSO_4_) complemented with bovine serum albumin (BSA) and harvested at stage 15. DMSO (dimethyl sulfoxide; Sigma) or SP600125 (Tocris), a JNK inhibitor, was added to the culture medium, from stage 11 to 15 at 0.5 μg.ml^−1^.

### RT-PCR

To determine *marveld3* expression, total RNA from 10 embryos was extracted for each developmental stage following the manufacturer instructions (RNeasy mini kit, Qiagen). Semi-quantitative PCR was performed for *Xenopus laevis marveld3* (Fw: ACAATGAGAGATTCTGTTACAGACGGGG and Rv: AAGCTCCCAACAGTTACAGCATCCATGGCT) and *gapdh* (Fw: ACTGCCACCCAGAAGAC and Rv: AAGTGCTTATTCCTTAGATG). Quantitative PCR was then performed with two pairs of primers for *marveld3* (Pair 1, Fw: GAGAGACCAAACCCATGAAG and Rv: GGTCTCCCTTTCTCTGTAGTA; Pair 2, Fw: CAAAGATCGTCACCAGTATCC and Rv: CAAGATTCCACCATCTGTCTC). *odc (ornithine decarboxylase)* was amplified as a normaliser (Fw: GGATGGATTTGTTACATCCGTC and Rv CACTCTCCGAGCTCACTTCCC).

### Whole-mount *in situ* hybridization (WISH), morpholinos, DNA

Embryos were processed for WISH as described in^39^. Control morpholino 5′-CCTCTTACCTCAgTTACAATTTATA-3′, a negative control targeting human a β-globin intron mutation responsible of β-thalassemia, and *Xenopus* MarvelD3 morpholinos (MD3A morpholino: AGACCCAAATCTTCCTTTTGTTCCC; MD3B morpholino: CCCGTCTGTAACAGAATCTCTCATT; Gene Tools; Fig. 1B) were used for the loss-of-function approach. 30 ng of MD3A or MD3B morpholino were injected for single morpholino experiments and 15ng of each when the two morpholinos were injected together. For the rescue experiments, either 2 ng of GFP tagged-full-length (*fl*) *marveld3* (BamH1/EcoR1; Fw: GTTCGCGGATCCGCCACCATGGTGAGCAAGGGCGAG; Rv: CTTCCGGAATTCTCAAACATAGTTGTTGGGTTTCTTTTTTAAC) or 750 pg of a mutated *marveld3* subcloned in pCS2+ (*7mut-marveld3*; BamH1/EcoR1; Fw: GTTCGCGGATCCGCCACCATGAGgGAcTCaGTaACtGAtGGcGAGAATCGGGCACCTAG; Rv: CTTCCGGAATTCTCAAACATAGTTGTTGGGTTTCTTTTTTAAC; mutated bases are in lower cases) were injected (Supplemental Fig. S1). Both *marveld3* constructs were based on *Xenopus laevis* sequences. The *dominant negative-jnk1* from *Xenopus* was a gift from Dr. Hidehiko Inomata previously described in^40^ and the *constitutively active-jnk* from Dr. Ira O. Daar was previously described in^41^. 200 pg of nuclear *β-galactosidase*, 500 pg of *dominant negative-jnk*, *constitutively active-jnk*, *chd* or *wnt8* mRNAs were injected. Following WISH and imaging, embryos were embedded in 1% agarose and transversal sections were generated with a blade.

### Cartilage staining and immunocytochemistry

Analysis of the cartilage morphology was performed on embryos (stage 45) fixed in MEMFA^39^, dehydrated, stained with 0.1 mg/ml Alcian blue 8GX (Sigma) (1 part acetic acid: 4 parts ethanol), cleared (70% ethanol + 1% HCl), prior to re-fixation and macerated in 2% KOH before dissection. To test morpholino efficiency, animal caps were harvested in Dent’s fixative (4 parts methanol: 1 part dimethyl sulfoxide), blocked in 2 mg/ml bovine serum albumin, 0.1% Triton X100 in phosphate buffered saline with 10% goat serum and incubated with polyclonal anti-xMarvelD3 (epitope amino-terminal amino acids: CTDGENRAPRKHRDHLENDT; Genosphere biotechnologies) and monoclonal anti-E-cadherin (610181; BD) antibodies. To analyse the activity of the JNK pathway, animal cap were fixed overnight in 4% paraformaldehyde (PFA) at 4 °C, permeabilised 15 minutes in PBT [2 mg/ml BSA, 0.1% Triton X100 in PBS], blocked 1 hour in PBT with 10% goat serum and incubated with polyclonal anti-p-c-Jun (S63; 9261 L, Cell Signaling) antibody followed by donkey anti-Cy3 labelled secondary antibody (Jackson Immunoresearch Inc.) combined to a Hoechst staining to visualize nuclei.

### Imaging

Embryos were imaged with a SMZ 1500 Nikon microscope (Apochromat HR Plan 0.5×/WD 136 mm objective; camera Digital Sight DS-Fi2; Nikon), and immunofluorescence images were obtained with a LSM710 confocal microscope (C-Apochromat 40×/1.2 N. A. objective; Zeiss) (Fig. 1D) or a Nikon Eclipse Ti microscope (20× S Plan fluor/0.45 N. A. objective; Nikon) (Fig. 8A–D; Supplemental Fig. S4A–D). All images were analysed with ImageJ.

### Image analysis, statistics and reproducibility

The intensity of the fluorescence for junctional MarvelD3 and E-cadherin (Fig. 1D) was measured with ImageJ. We quantify the number of cell with positive nuclear p-c-Jun by overlapping the nuclei and the phospho-antibody images and manually counted the phospho-antibody staining positive nuclei and calibrate it to the total number of nuclei. Values were then normalized to the control as indicated in the legends. Error bars indicate standard errors of the mean; ANOVA was used to analyse experiments with more than 2 groups; exact p values obtained from two tailed tests (Mann Whitney or t-tests as indicated in the legends) are indicated in the graphs; quantifications are based on at least three independent experiments. The numbers at the bottom right of the pictures correspond to the number of embryos or animal caps presenting the main observed phenotype. The numbers on/under the graphs correspond to the number of embryos, animal caps or cells counted as specified in the legends and are derived from at least three repeats. Morpholino experiments and corresponding recues were performed in parallel for each type of analysis.

### Data availability

All data supporting the conclusions here are available from the authors on reasonable request.

## Electronic supplementary material

Supplementary figures
