# Supplementary material for: Control of neural crest induction by MarvelD3-mediated attenuation of JNK signalling
Source: Sci Rep. 2018 Jan 19;8:1204. doi: 10.1038/s41598-018-19579-5 (PMC5775312; doi:10.1038/s41598-018-19579-5)
Supplement: Supplementary file 1 — Supplementary figures [file 41598_2018_19579_MOESM1_ESM.pdf]

Supplemental Figures

Control of neural crest induction by MarvelD3-mediated attenuation of JNK signalling

Barbara Vacca[1], Elena Sanchez-Heras[1], Emily Steed[1,4], Sophie L. Busson[1], Maria S. Balda[1], Shin-Ichi Ohnuma[1], Noriaki Sasai[2], Roberto Mayor[3] and Karl Matter[1]

[1]Institute of Ophthalmology, University College London, London, EC1V 9EL, UK.

[2]Developmental Biomedical Science, Graduate School of Biological Sciences, Nara Institute of Science and Technology (NAIST), 8916-5, Takayama-cho, Ikoma 630-0192, Japan.

[3] Department of Cell and Developmental Biology, University College London, Gower Street, London, WC1E 6BT, UK.

[4] Present address: Institute of Epigenetics and Stem Cells, Helmholtz Zentrum München, D-81377, Munich, Germany.

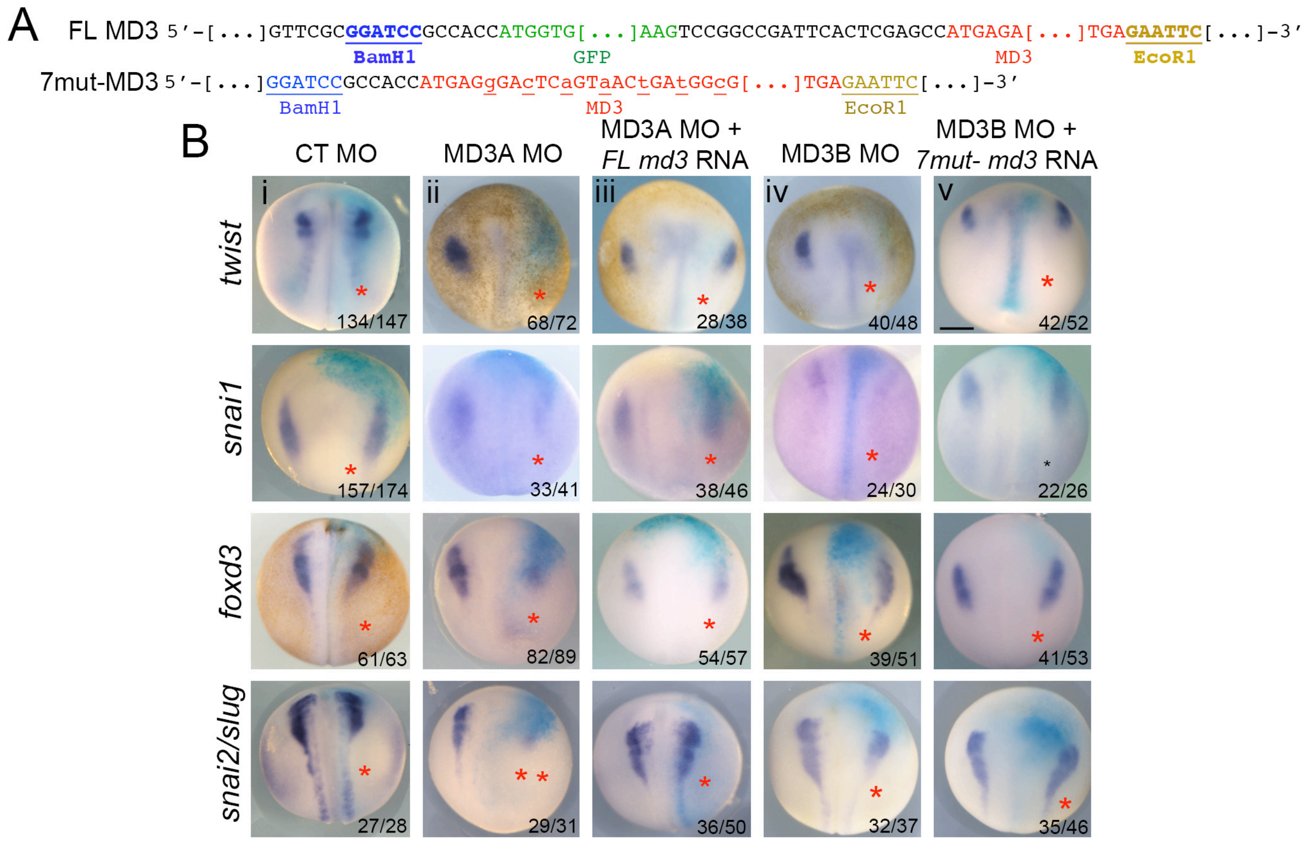

Supplemental Figure S1. Depletion of MarvelD3 and neural crest development. (A) *gfp-fl marveld3* was inserted in pCS2+ vector (BamH1/EcoR1). *7mut-marveld3* was subcloned in pCS2+ vector (BamH1/ EcoR1). Restriction sites: underlined; mutated bases of *marveld3*: underlined lower cases. (B) neural crest induction (stage 15) was determined by WISH for *twist*, *snai1*, *foxd3* and *snai2/slug* in control morpholino- (i), MD3A morpholino- (ii), MD3B morpholino-injected embryos (iv) and embryos co-injected with *FL marveld3* (iii) or *7mut-marveld3* mRNA (v), respectively; the number of embryos analysed is indicated at the bottom right of each panel; red asterisk, injected side of the embryo with light blue β-galactosidase staining; purple corresponds to the WISH staining; scale bar, 500 μm.

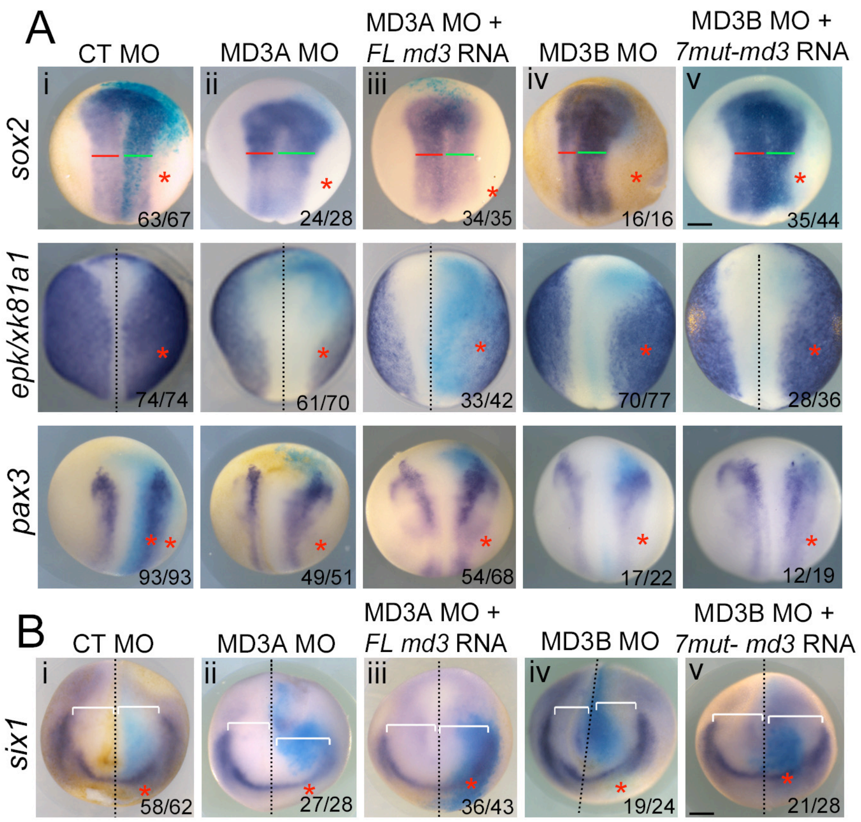

Supplemental Figure S2. Effect of MarvelD3 depletion on neural plate border specification. (A, B) Neural plate border specification was established by WISH against *sox2*, *epk/xk81a1*, *pax3* and *six1* in control morpholino- (A, i; B, i), MD3A morpholino- (A, ii; B, ii), MD3B morpholino-injected embryos (A, iv; B, iv) and embryos co-injected with fl *marveld3* (A, iii; B, iii) or *7mut-marveld3* mRNA (A, v; B, v) at stage 15. Scale bars, 500 µm; (A) red and green bars demarcate the medio-lateral expansion of *sox2*; (A, B) black dotted line identify the midline; (B) white bracket indicate the distance from the midline; the number of embryos analysed is indicated at the bottom right of each panel; red asterisk, injected side of the embryo with light blue β-galactosidase staining. Scale bars, 500 µm; red and green bars demarcate the medio-lateral expansion of *sox2*; black dotted line identify the midline; the number of embryos analysed is indicated at the bottom right of each panel; red asterisk, injected side of the embryo with light blue β-galactosidase staining; purple staining corresponds to WISH.

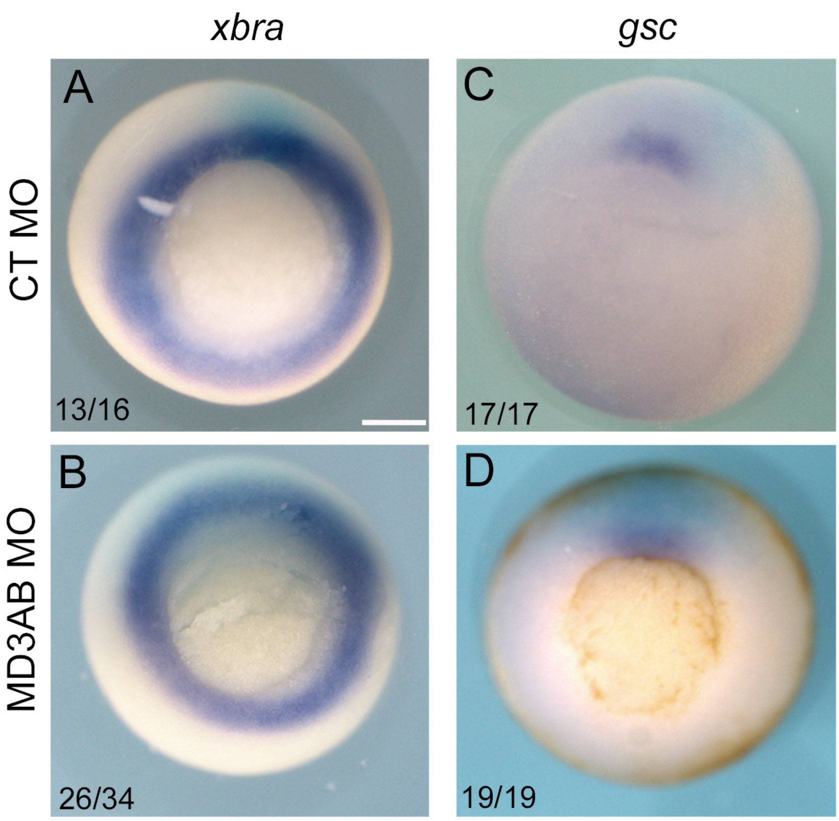

Supplemental Figure S3. MarvelD3 depletion and mesoderm formation. Effect of MD3AB morpholinos on the mesoderm was analysed by WISH against *xbrachyury* (*xbra*; A, B) and *goosecoid* (*gsc*; C, D) at stage 11; ventral view. Scale bar, 500 µm; the number of embryos analysed is indicated at the bottom left of each panel; light blue indicates the β-galactosidase staining on the injected side of the embryo while purple corresponds to WISH staining.

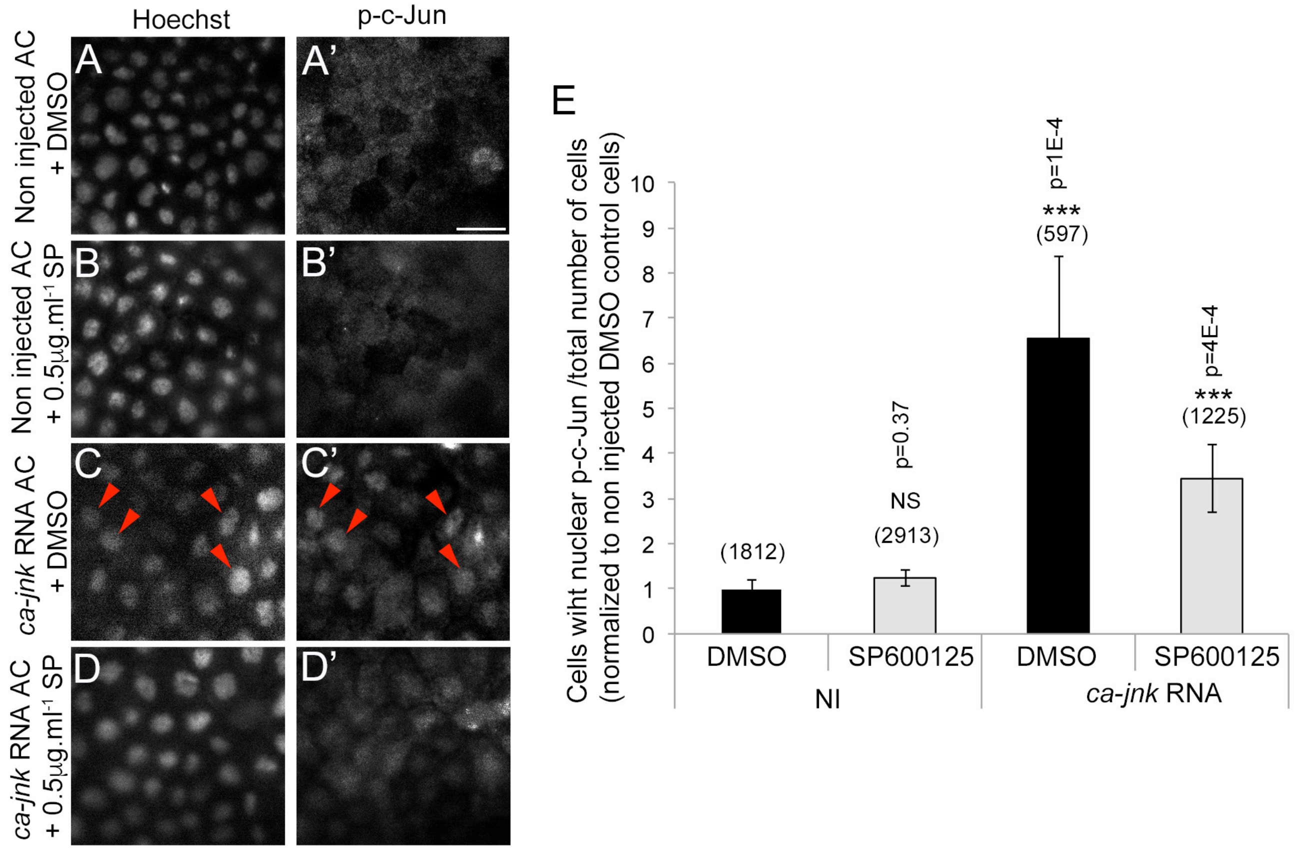

Supplemental Figure S4. JNK inhibitor efficiency in *Xenopus*. Analysis of SP600125 activity by immunofluorescence for p-c-Jun in non-injected (A, B) or with *constitutively active-jnk* mRNA-injected animal caps (C, D) treated from stage 11 to 15 with DMSO (A, C) or 0,5µg.ml^{-1} SP600125 (B, D). Nuclei were stained with Hoechst; red arrowheads: examples of nuclear co-localization between Hoechst and p-c-Jun staining; scale bar, 20 µm. (E) Quantification of the number of cells with a positive nuclear p-c-Jun staining / total number of cells. Mann-Whitney test p values and the total of cells counted (numbers in brackets) are indicated; the values are normalized to NI + DMSO; black bars, DMSO treated animal caps; grey bars, SP600125 treated animal caps.

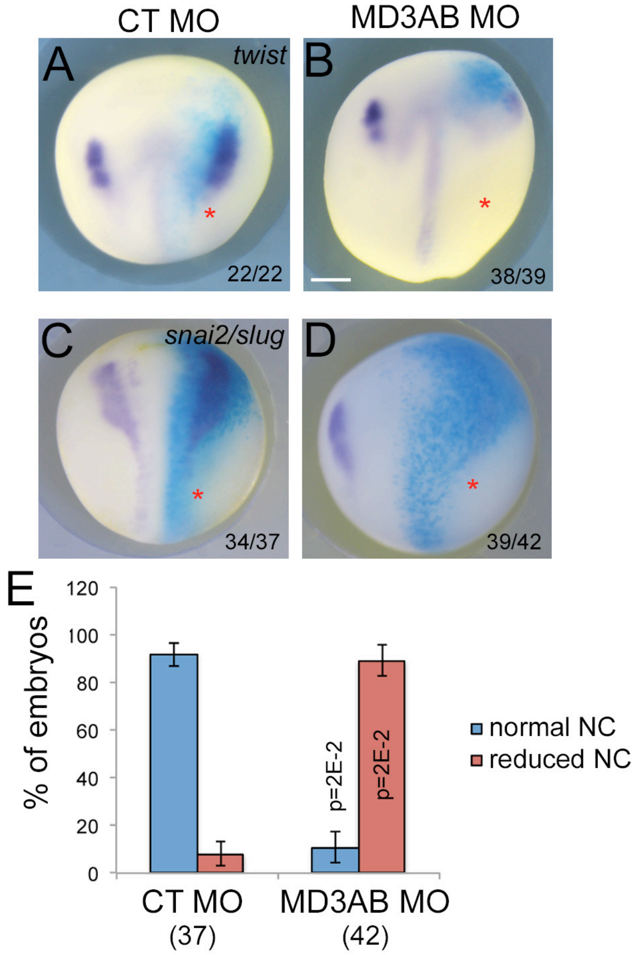

Supplemental Figure S5. Morpholino control experiments for JNK pathway experiments in figure 10. WISH against *twist* (A, B) and *snai2/slug* (C, D) in control or MD3AB morpholino-injected embryos, corresponding controls for Fig. 10A-D and Fig. 10E-H, respectively. Scale bar, 500 μm. (E) Quantification of the neural crest phenotype through *snai2/slug* expression for (C, D). The number of embryos analysed is indicated at the bottom right of each panel or under the graph; red asterisk, injected side of the embryo with light blue β-galactosidase staining. Mann-Whitney test p values are indicated; blue bar, normal neural crest; red bar, reduced neural crest.
